# Pulmonary metastases from mucinous colorectal cancers and their appearance on CT: a case series

**DOI:** 10.1259/bjrcr.20220102

**Published:** 2022-11-01

**Authors:** Jonathan Ian Fairburn Jackson, Iain T H Au-Yong, Yutaro Higashi, Rafael Silverman, Christopher G D Clarke

**Affiliations:** 1 Department of Radiology, Nottingham University Hospitals NHS Trust, Nottingham, United Kingdom; 2 Department of Oncology, Nottingham University Hospitals NHS Trust, Nottingham, United Kingdom; 3 Department of Radiology, Nottingham University Hospitals NHS Trust and Honorary (Clinical) Assistant Professor, University of Nottingham School of Medicine (Orcid ID 0000-0002-8092-9877), Nottingham, United Kingdom

## Abstract

Mucinous colorectal adenocarcinoma represents a small proportion of all colorectal cancers, characterised by mucinous tumour components. While its pattern of metastatic spread differs from that of conventional colorectal adenocarcinoma, pulmonary metastases are commonly seen in both mucinous and non-mucinous types.

The assessment of pulmonary nodules in the context of malignancy is a commonly encountered problem for the radiologist given the high prevalence of benign pulmonary lesions. Low density of a pulmonary nodule on CT evaluation is one of the recognised and well-documented features of benignity that is used in the radiological assessment of such nodules.

We present three cases of patients with histologically proven mucinous colorectal adenocarcinoma with evidence of pulmonary metastases. In all cases, the metastases were of low density on CT and in one case were initially suspected to represent benign hamartomatous lesions.

There has been little documented about the density of mucinous pulmonary metastases on CT. We suspect the low density seen in the metastases in each case is accounted for by their high internal mucinous components.

The cases presented here demonstrate the importance of recognising that mucinous colorectal metastases can be of low density and therefore mimic benign pathology. This review may help the radiologist to consider shorter interval follow-up of such lesions in the context of known mucinous neoplasms, or to investigate for an extrathoracic mucinous carcinoma in the presence of multiple low-density pulmonary nodules.

## Introduction

Mucinous adenocarcinoma accounts for 10% of all colorectal cancers and is defined as a colonic adenocarcinoma that is made up predominantly of extracellular mucin.^1–3^ In comparison to its more common, non-mucinous counterpart, the tumour is often right-sided and is generally recognised to have a poorer outcome.^1–3^ The diagnosis of the primary tumour can often be challenging, as biopsy results are frequently indeterminate due to the paucity of cellular material obtained. In comparison to non-mucinous colonic adenocarcinoma, which primarily metastasises to the liver, peritoneal metastases are more likely with mucinous cancers.^4,5^ Both, however, can metastasise to the lungs.^4,5^

The general consensus is that in the case of pulmonary lesions, a lower Hounsfield unit (HU) value on CT imaging is associated with an increased likelihood of benignity.^6–8^ There has been little description of the appearance of mucinous pulmonary metastases in the literature to date, with a limited number of case reports demonstrating pulmonary metastases from primary mucinous colorectal cancers to be of low density.^9–11^ We present three cases of mucinous colorectal cancers in patients whose CT imaging demonstrated concurrent mucinous pulmonary metastases.

## Cases

### Case 1

**Figure 1.**
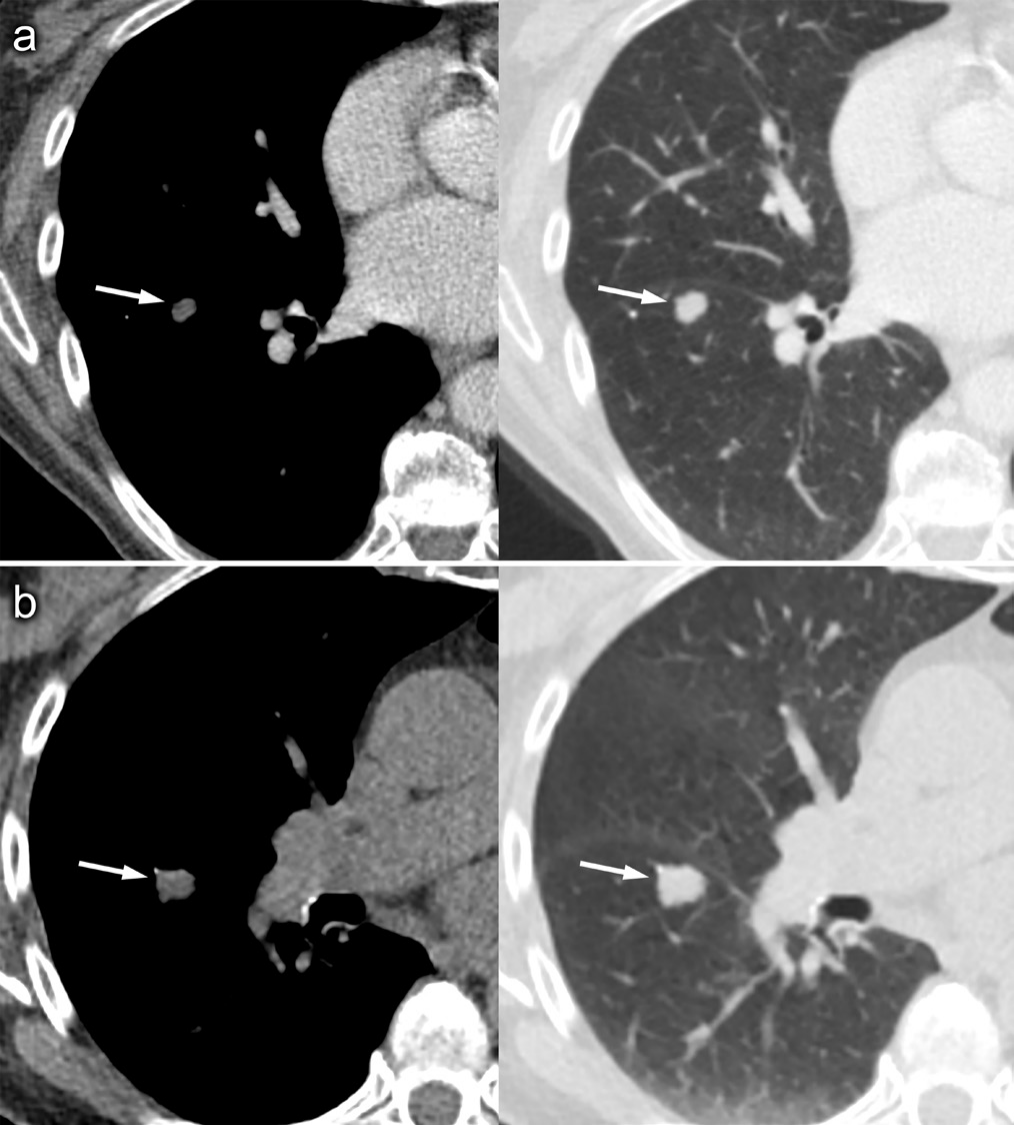
Axial CT images on soft-tissue and lung window settings on post-contrast CT (A) and the CT component of a non-contrast PET-CT (B) performed 6 weeks later, which show an enlarging low-density pulmonary nodule within the lower lobe of the right lung. Note that image A suggests the presence of macroscopic fat anteriorly within the lesion. PET, positron emission tomography.

A 78-year-old female who had 12 months previously undergone resection of a histologically proven mucinous rectal adenocarcinoma had a follow-up contrast-enhanced CT due to a persistently mildly elevated serum carcinoembryonic antigen (CEA), which demonstrated a low-density, irregular 10 mm nodule in the right lower lobe, averaging −14 HU on region of interest (ROI) measurements (Figure 1). The ROI measurement (for this and the other cases) was calculated by placing a circular ROI incorporating as much of the overall nodule as possible. This nodule was new since the staging CT performed 12 months previously. There was also the impression of macroscopic fat within it anteriorly. This showed a mild increase in size to 12 mm on subsequent positron emission tomography (PET) performed 6 weeks later and was not avid, demonstrating an SUVmax below mediastinal blood pool. The patient underwent a video-assisted thoracoscopic surgery (VATS) resection of the lesion, which was confirmed histologically to be a mucinous colorectal carcinoma metastasis.

### Case 2

**Figure 2.**
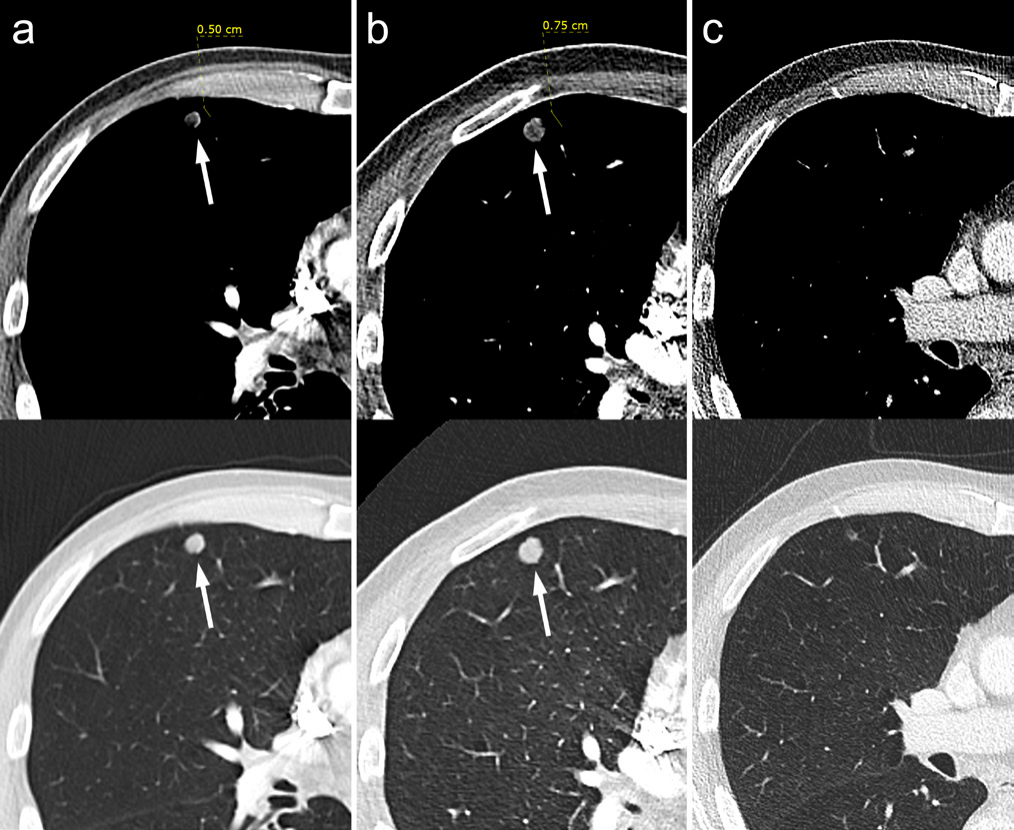
Axial CT images on soft-tissue and lung window settings demonstrating one of multiple low-density pulmonary nodules within the right lung on initial arterial-phase contrast-enhanced CT (A), which increases in size on a subsequent CTPA (**b**) and then can be seen to have almost completely resolved following chemotherapy treatment on subsequent restaging contrast-enhanced CT (C). CTPA, CT pulmonary angiography.

A 69-year-old male underwent CT colonography for further assessment of an obstructing rectosigmoid tumour detected at colonoscopy. Concurrent post-contrast CT of the chest revealed left upper lobe and right middle lobe rounded lesions measuring up to 12 mm, initially suspected to represent hamartomas due to their low density (right middle lobe nodule measured −45 HU, see Figure 2). Biopsies of the rectosigmoid lesion performed at initial colonoscopy and then at two subsequent sigmoidoscopies revealed tubovillous adenoma with high-grade dysplasia only, without features of malignancy. Rectal MRI however demonstrated T2-hyperintense components suggestive of mucin within the primary tumour with extension beyond the muscularis propria and multiple enlarged local mesorectal lymph nodes. The patient underwent a Hartmann’s procedure and a diagnosis of adenocarcinoma was obtained from resected tissue. A subsequent CT pulmonary angiogram (CTPA) performed 2 months after the initial CT colonoscopy showed that the pulmonary lesions had increased in size. The left upper lobe nodule measured 15 mm, previously 12 mm (initial volume 1.12 cm^3^, final volume 1.787 cm^3^, volume doubling time (VDT) = 102 days, suggesting malignancy), and the right middle lobe nodule measured 383 mm^3^, previously 143 mm^3^, VDT 49 days, in keeping with metastatic disease. These initially responded well to palliative chemotherapy, with many lesions resolving, although subsequent CT demonstrates disease progression with increase in size of a low density lingular metastasis (average −14 HU).

### Case 3

**Figure 3.**
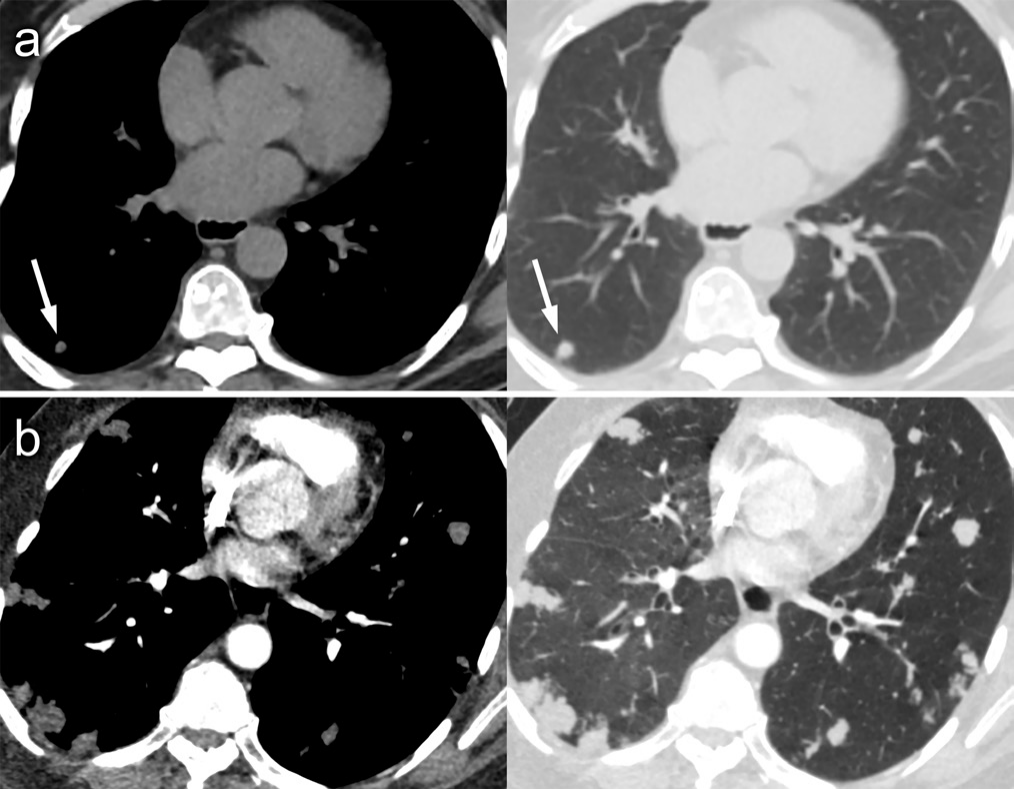
Axial CT images on soft-tissue and lung window settings demonstrating one of several small low-density pulmonary nodules within the right lower lobe on the CT component of a non-contrast PET-CT (arrowed) with a tiny focus of low density to its left side which may represent macroscopic fat (A). There is evidence of marked disease progression on subsequent CTPA performed 11 months later, with significant increase of the index nodule and several new lesions (B). CTPA, CT pulmonary angiography; PET, positron emission tomography.

A 57-year-old male with a history of right hemicolectomy 9 years previously for a mucinous colorectal cancer (CT of the chest at that time was clear) underwent a CT-guided biopsy for a right psoas mass, the histology for which demonstrated a mucinous colonic adenocarcinoma metastasis. A subsequent staging PET-CT scan demonstrated multiple small rounded pulmonary lesions of low density, in keeping with pulmonary metastases. A nodule in the right lower lobe measured 5 mm in diameter and −29 HU in density (Figure 3). The nodules increased in size and number on subsequent CTPA performed 1 year later suggesting progressive disease, the index nodule in the right lower lobe now measuring 13 mm in diameter. The lesions remained of low attenuation on this subsequent study (0 to + 20 HU).

## Discussion

Mucinous adenocarcinoma accounts for 10% of all colorectal cancers, defined as a malignant tumour that is predominantly made up of extracellular mucin.^1^ Mucinous colorectal adenocarcinoma are a subset of tumours demonstrating differing biological behaviour even when traditional prognostic factors are accounted for.^2^ In comparison to their more common, non-mucinous counterpart, these tumours often arise from the proximal colon, have a female preponderance, and are generally considered to have a poorer outcome.^3^

The metastatic spread of mucinous colorectal cancer is predominantly peritoneal, in comparison to non-mucinous adenocarcinoma for which hepatic metastases are more common.^4,5^ Pulmonary metastases occur in both cancer types.^5^ The attenuation values of solitary pulmonary nodules have been extensively researched, reflecting the common radiological dilemma of attempting to discern whether a pulmonary nodule is benign or malignant. While it has been well-documented that a lower density solitary pulmonary nodule confers a reduced likelihood of malignancy (with a mean HU measurement of less than 15 HU strongly correlating with benignity), the literature regarding the measured attenuation and enhancement of multiple lesions is much sparser.^6–8^

Furthermore, there exist multiple reports of malignant pulmonary lesions which can be of low density, including certain primary lung tumours and lesions that have undergone necrosis or cystic change, and cystic metastases from ovarian or gastric cancers. A case report by Yousem et al. (1986) is one of the few that includes HU values in its description of pulmonary metastases.^9^ It describes cases of thoracic metastases from testicular tumours with values of between −8 and +15 HU on contrast-enhanced CT, concluding that low-density pulmonary or mediastinal lesions should prompt further investigation in appropriate patients to investigate for an extrathoracic primary malignancy.

Despite this, the HU measurements of such lesions are seldom described, with only one case report found in the literature relating to mucinous tumours, which is worthy of brief discussion. Miyake et al. (1995) describe two cases of mucinous lung lesions with attenuation values of between 4 and 8 HU on pre-contrast CT, enhancing to between 21 and 26 HU on post-contrast imaging.^10^ One of these was a metastatic mucinous lesion from a primary mucinous colon cancer. Both pre- and post-contrast CT examinations were performed in each case, allowing the authors to compare HU measurements in both non-enhanced and enhanced studies. They concluded that low-attenuation pulmonary lesions measuring less than 10 HU with slight contrast enhancement on post-contrast imaging and irregular margins were features in keeping with primary or metastatic mucin-producing tumours. Although our data lack the dual-phase imaging acquired in this case series, the mean HU values correlate with those cited in the above cases.

The British Thoracic Society guidance and Fleischner Society guidance^12,13^ are widely employed for the management of pulmonary nodules. These guidelines recommend that nodules which demonstrate evidence of macroscopic fat within them suggesting the diagnosis of a hamartoma can be considered benign and may not require further investigation or surveillance. In practice, the appearances of hamartomas can be quite diverse, and not all hamartomas contain fat or the characteristic popcorn calcification.^14^ Knowledge that mucinous metastases can be of low attenuation mimicking fat density is useful in the initial interpretation of such nodules, in that it should not necessarily be assumed that such nodules are benign and a lower threshold for surveillance may be required. This appearance may also prompt patient assessment for a mucinous tumour, especially if the patient has symptoms.

## Conclusion

We present a selection of cases of proven mucinous colorectal cancer with concurrent pulmonary metastasis. The striking feature of the pulmonary metastases in these cases is their low-density appearances on CT imaging, which is a feature seldom described in the literature to date and one that may lead the reporting radiologist to dismiss them as benign pulmonary nodules. We conclude that although uncommon, low-density pulmonary metastases can occur in mucinous colorectal cancers and that multiple low-attenuation pulmonary lesions may be used to prompt further investigation for an extrathoracic primary mucinous tumour.

## Learning points

- Pulmonary metastases of mucinous colorectal cancer origin can be of low density on CT and mimic benign lesions.
- Hamartoma is an important benign differential diagnosis. The presence of macroscopic fat and/or diffuse central, laminated or popcorn calcification is considered suggestive of this diagnosis.
- Caution should be exercised in dismissing pulmonary nodules as benign solely on the basis of low density on CT, particularly in the presence of a known mucinous colorectal tumour.
- The presence of low-density pulmonary nodules may prompt consideration of the presence an extrathoracic mucinous primary tumour.
